# MicroRNAs and the Genetic Nexus of Brain Aging, Neuroinflammation, Neurodegeneration, and Brain Trauma

**DOI:** 10.14336/AD.2018.0409

**Published:** 2019-04-01

**Authors:** Saumyendra N. Sarkar, Ashley E. Russell, Elizabeth B. Engler-Chiurazzi, Keyana N. Porter, James W. Simpkins

**Affiliations:** Center for Basic and Translational Stroke Research, Rockefeller Neuroscience Institute, West Virginia University, Morgantown, WV 26506, USA; Center for Basic and Translational Stroke Research, Rockefeller Neuroscience Institute, West Virginia University, Morgantown, WV 26506, USA

**Keywords:** aging, microRNAs, brain, neuroinflammation, neurodegeneration, inflammaging

## Abstract

Aging is a complex and integrated gradual deterioration of cellular activities in specific organs of the body, which is associated with increased mortality. This deterioration is the primary risk factor for major human pathologies, including cancer, diabetes, cardiovascular disorders, neurovascular disorders, and neurodegenerative diseases. There are nine tentative hallmarks of aging. In addition, several of these hallmarks are increasingly being associated with acute brain injury conditions. In this review, we consider the genes and their functional pathways involved in brain aging as a means of developing new strategies for therapies targeted to the neuropathological processes themselves, but also as targets for many age-related brain diseases. A single microRNA (miR), which is a short, non-coding RNA species, has the potential for targeting many genes simultaneously and, like practically all other cellular processes, genes associated with many features of brain aging and injury are regulated by miRs. We highlight how certain miRs can mediate deregulation of genes involved in neuroinflammation, acute neuronal injury and chronic neurodegenerative diseases. Finally, we review the recent progress in the development of effective strategies to block specific miR functions and discuss future approaches with the prediction that anti-miR drugs may soon be used in the clinic.

Aging is a complex and integrated gradual deterioration of cellular activities in organs of the body that corresponds with increased morbidity and mortality [1]. Various tissues experience progressive, functional decreases over time, which manifest as age-related phenotypes and diseases, including cancer, diabetes, cardiovascular disorders, neurovascular disorders, and neuro-degenerative diseases [2-6]. This review outlines some of the important hallmarks of aging, together with examples of specific miRs that are known to be involved in each specifically, as well as aging in general (Fig. 1). There are nine tentative “hallmarks” of aging, as recently described by Lopez-Otin et al. [1]. These hallmarks include (i) gradual deterioration in repairing damaged DNA, (ii) altered nutrient signaling, (iii) loss of protein homeostasis, (iv) mitochondrial dysfunction, (v) stem cell exhaustion, (vi) telomere shortening, (vii) dysregulation of the genes by epigenetic and mRNA processing changes, (viii) premature cellular senescence, and (ix) altered intra-cellular communication.

The implications for heterochronic (young-old) parabiosis experimental findings in mice are profound; circulating factors can inhibit the aging phenotype by influencing the resident tissues, which have the inherent potential to regenerate and function analogous to juvenile tissues [7-10]. Systemic administration of plasma derived from juvenile to aged mice reverses many aging phenotypes including cardiac hypertrophy [8], synaptic plasticity dysfunction and cognitive deficits [10], muscle degeneration [9], and hypo-perfusion of the brain [7]. GDF11, a member of the transforming growth factor beta (TGF-β) superfamily, is a circulating factor that declines with age, and may be responsible for some of these effects, as it has been shown to reduce age-related cardiac hypertrophy [9]. Further, exposing young mice to plasma from old mice decreases synaptic plasticity, and impairs contextual fear conditioning, spatial learning, and memory [11]. A candidate circulating factor in aged murine plasma is C-C Motif Chemokine Ligand 11, (CCL11), a chemokine gene family member. It has been reported that the levels of CCL11 are increased in the plasma and cerebrospinal fluid of healthy aging humans [11]. Thus, the systemic immune related factor, CCL11, is a potentially critical contributor to the susceptibility of the aging brain to cognitive impairments.

The factors regulating the expression of GDF11 and CCL11, the tissues involved in their production, their mechanism(s) of action, and why their expression changes during aging remains yet to be determined. Besides proteins, other molecules, such as RNA and exosomes containing miRs can induce or inhibit the aging phenotypes [12]. Molecular components that govern both the structure and function of vital organs in the body are encoded in the genome and regulated by the expression of various genes. As a consequence, changes in regulation of gene expression during aging might be directly related to the expression of the aging phenotype, and age-related pathology in a specific organ. Transcriptional profiling of single cells, including neurons, microglia, astrocytes, oligodendrocytes, and endothelial cells, by specific RNA sequencing analysis of human prefrontal cortex of subjects ranging in age from 26 to 106 years, defines two sets of aging-related changes in gene expression [13, 14].

First, one set show reduced expression, while a second set of genes show increased expression, after age 40. According to Gene Ontology (GO) and Kyoto Encyclopedia of Genes and Genomes (KEGG) enriched score categories, the first sets of genes (reduced expression) play a central role in grey and white matter plasticity, mitochondrial function, learning and memory, intracellular transport, oxidative and cellular stress resistance, DNA repair enzymes, epigenetic and transcription processes. The second set of genes (increased expression) is categorized as neuroinflammation (inflammaging) genes. GO and KREGG analyses of expression profiles of miRs in the aging cerebellum and cortex of both chimpanzees and human have suggested that upregulated miR target genes are factors in cognitive decline and neurodegenerative disorders during aging [15].

**Figure 1.**
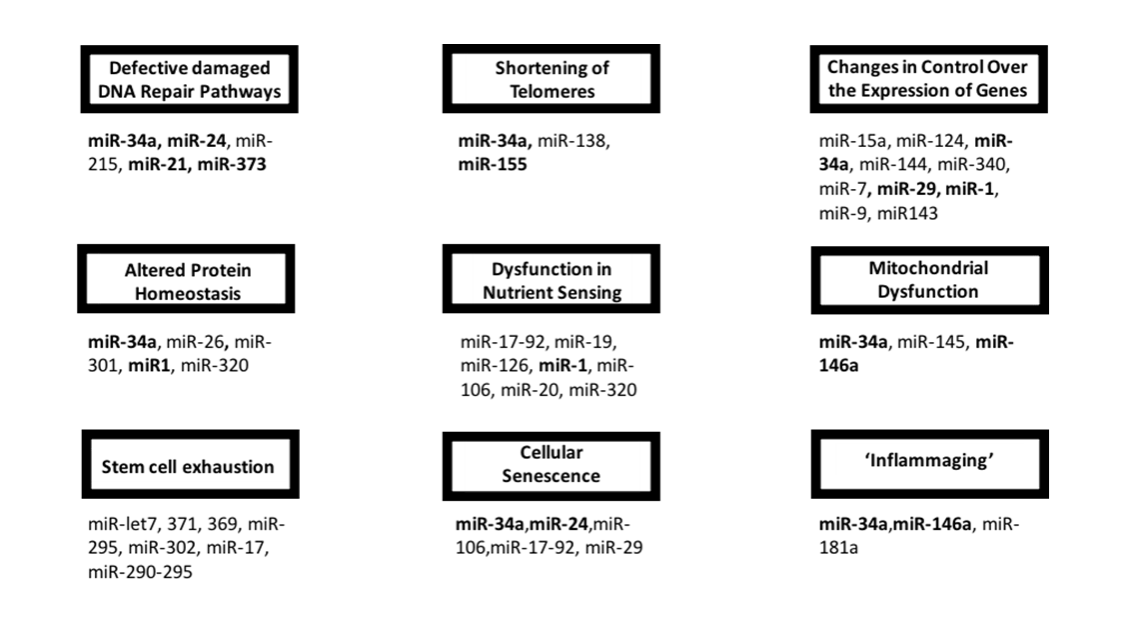
The hallmarks of mammalian aging and the miRs that target genetic networks involving these pathways. Associated miRs with each of the hallmark of aging are indicated. The miRs involving more than one aspect of aging are bolded.

**Figure 2.**
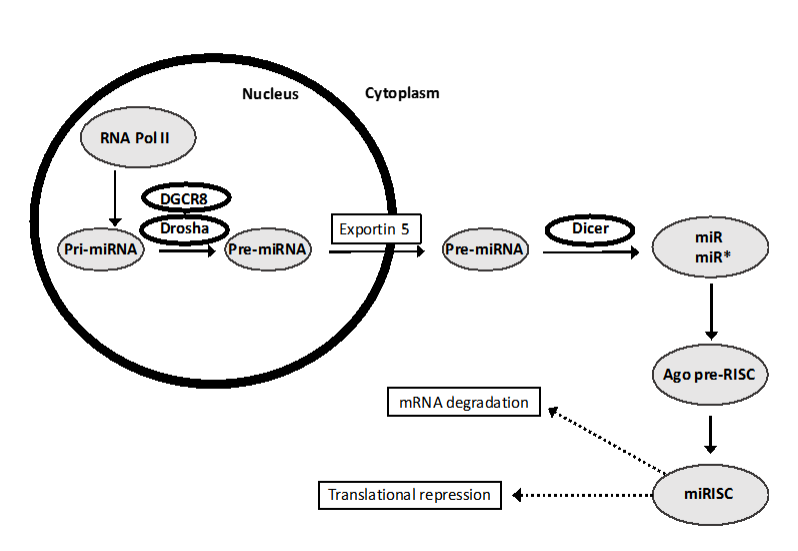
miR Biogenesis and Post-transcriptional Gene Regulation.

One of the main reasons for changes in gene expression during aging is epigenetic regulation. This includes alterations in the methylated states of regulatory DNA sequences, covalent modifications of histone proteins, and the expression of regulatory non-coding RNAs, such as miRs. Presently, thousands of miRs have been identified in plants and mammals, and as of today, over 1881 human miRs have been reported in miRBase [http://www.mirbase.org/; 16]. It is important to note that miRBase miRNAs are only sequence based, and many of them are yet to be functionally validated. As such, the aforementioned number of putative human miRNAs is likely an over estimate. miRs have recently emerged as important regulators of cellular senescence and aging. Several reviews describing miRs and their role in the aging process have been published [17, 18].

The cellular and molecular hallmarks of aging have been categorized [1], and the present review assesses many of the candidate hallmarks of aging, together with examples of specific miRs that are known to be involved in aging. Here we highlight how certain miRs can mediate dysregulation of genes involved in aging, which are also linked with aging-related diseases of the brain. Further, we describe the role of miRs in the brain’s inflammatory response during aging and age-related diseases. Finally, we consider the role of miRs in acute brain damage from stroke, as well as neurodegenerative diseases.

## miRs: Genomics, Biogenesis, and Function

miRs are small, endogenous ~22 nucleotide RNAs whose biogenesis occurs in all mammalian and plant cells and can play important regulatory roles by targeting mRNAs for cleavage or translational repression. Many miR genes are clustered in the same region of a chromosome, and some miRs have homologues; e.g. miR-34 is a family of three (miR-34a, -34b, and -34c); the reader is referred to the GeneCards website (http://www.genecards.org).

miR genes are transcribed by RNA pol II or pol III as long precursors (pri-miRs) that are then cleaved into smaller RNAs (pre-miRs) by the microprocessor in the nucleus. Pre-miRs are then exported by exportin 5 into the cytoplasm, where the enzyme Dicer cleaves them to produce short double stranded RNA molecules. Of the two strands, one is generally referred to as the primary, or mature miR sequence, while the other is known as the passenger strand (denoted as miR*) [19]. The mature miR sequence is eventually loaded into the RNA Induced Silencing Complex (RISC) and guides the complex in mRNA target binding by base pairing. The passenger strand is usually degraded, however some evidence suggests that it can also interact with RISC and exert regulatory roles [19]. By pairing with target mRNAs, miRs are able to downregulate gene expression by targeting mRNA for degradation or transcriptional repression (Fig. 2).

### Defective Damaged DNA Repair Pathways

Cells continuously encounter various DNA-damaging factors, which give rise to DNA damage throughout life. Cells remove endogenous and exogenous sources of DNA damage through their conserved DNA repair and cell cycle checkpoint pathways, which help cells to either maintain genomic stability, or prevent cells from entering mitosis [20]. Multiple DNA repair pathways have evolved to resolve various DNA lesions. These pathways include base excision repair (BER)/single strand break repair (SSBR), homologous recombination repair (HR), non-homologous end-joining repair (NHEJ), nucleotide mismatch repair (NMR), translesion DNA synthesis (TLS), and telomere repair [21]. Several studies have shown that expression of DNA repair enzymes decreases during aging. One possible mechanism by which this reduction of enzymes occurs could be that aging induces miR expression, which targets repair genes, and represses their translation.

**Figure 3.**
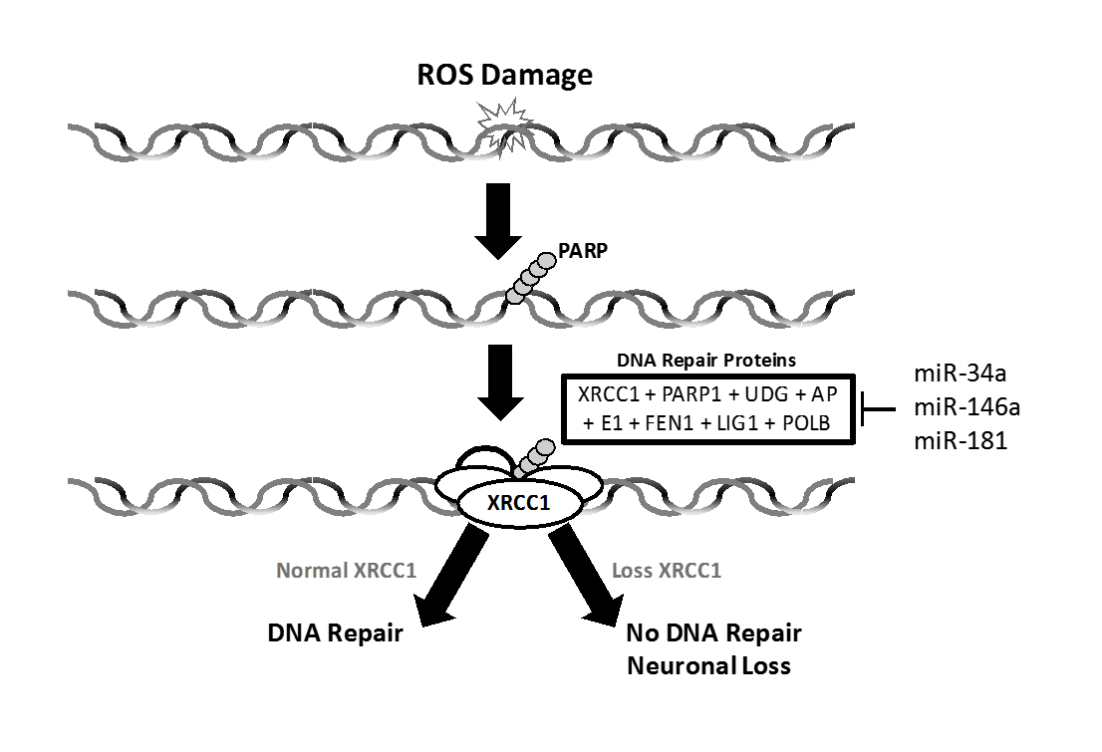
MicroRNAs Target Enzyme-Scaffold Complex. Chains of molecules along with XRCC1 scaffolding protein recognize ROS induced DNA damage and recruit six enzymes to form the BER/SSBR-scaffold complex. MicroRNAs shown are identified by in silico analysis and target many components of this complex.

There are many excellent reviews published on the role of miRs and exogenous DNA damage responses in proliferating cells, but the role of miRs and exogenous DNA damage in terminally differentiated cells has not been extensively reviewed. Defective repair of oxidized DNA has been noted in the brains of patients with Alzheimer’s disease (AD), and elevated levels of oxidized DNA bases were observed in the cerebrospinal fluid of AD patients. The DNA repair mechanism BER/SSBR is the predominant mechanism for the removal of oxidized DNA bases and oxidized DNA break termini, which are formed at high frequency in neuronal cells of the adult brain. BER/SSBR proceeds through five steps, involving six enzymes, including poly-(ADP ribose) polymerase (PARP1), uracil DNA glycosylase (UDG), Apurinic/ apyrimidine endonuclease (APE1), DNA polymerase beta (POLB), DNA ligase1 (LIG1), flap structure-specific endonuclease (FEN1) and the scaffold protein, XRCC1, which plays a key role in coordinating BER/SSBR complex formation (Fig. 3). In humans, a function mutation in XRCC1 results in a phenotype that is characterized by neurodegeneration in the cerebellum and basal ganglia [22]. Moreover, neural specific loss of XRCC1 in mice leads to abnormal hippocampal function [23]. Experimental evidence linking targets of various miRs that are induced during aging with BER/SSBR is scant. In silico analysis (Sarkar, unpublished observation) revealed that miR-34a, whose expression is known to be increased in the aging heart and brain, targets XRCC1, FEN1, and UDG. Additional miRs target BER/SSBR-associated enzymes and proteins: miR-181 targets PARP1; miR-146b targets XRCC1 (Fig. 3).

### miRs and Deregulated Nutrient Sensing

**Figure 4.**
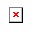

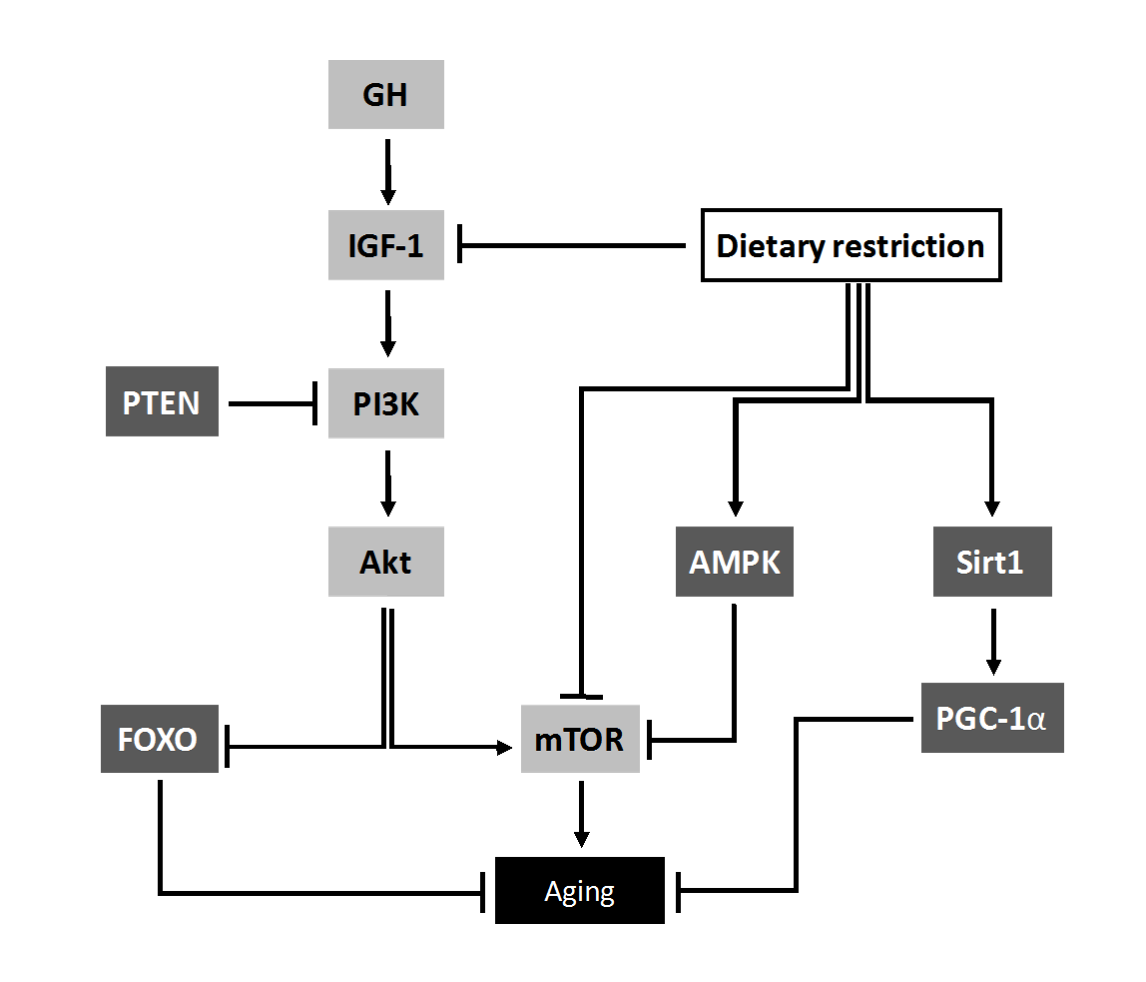
miRs involved in deregulation of nutrient sensing pathway. Schematic diagram showing the growth hormone (GH) and insulin growth factor 1 (IGF-1) signaling pathway and its association with dietary restriction and aging. GH = Growth Hormone, PTEN = Phosphatase and Tensin homolog, PI3K = Phosphatidylinositol-4,5-bisphosphate 3-kinase, AKT = Protein kinase B, AMPK = AMP-activated protein kinase, Sirt1 = sirtuin (silent mating type information regulation 2 homolog) 1, mTOR = mechanistic target of rapamycin, PGC-1α = Peroxisome proliferator-activated receptor gamma coactivator 1-alpha, FOXO = FOXO family of Forkhead transcription factors.

Deregulated nutrient sensing is a hallmark of aging. The intracellular signaling pathway of insulin-like growth factor (IGF-1)/insulin and downstream intracellular effectors such as AKT, mTOR, and FOXO are the most conserved aging-controlling pathways in evolution [24]. Age-specific profiling of miRs in skeletal muscle identified miR-126 as a regulator of muscle plasticity, and an inhibitor of IGF-1 signaling [25]. Other published work has shown that upregulation of miR-190b plays a role in decreasing IGF-1, leading to the induction of insulin resistance [26]. Another miR, miR-1, targets the IGF-1 signal transduction pathways in both cardiac and skeletal muscles [27]. Several other studies have also reported that multiple miRs regulate the components of nutrient sensing pathways, such as miR-1, miR-320, and miR-206 by targeting IGF-1 [28] and miR-216a, miR-217, and miR-21 by targeting phosphatase and tensin homolog (PTEN) [29]. Further, there are reports showing that several miRs regulate the expression of SIRT1, an ortholog of Sir2, which is implicated in the regulation of life span, stress resistance, and metabolism [30]. Additionally, the expression of miR-217 increases during aging and targets SIRT1 in endothelial cells [31]. miR-34a, has also been found to target SIRT1 [32], implying that there might be a connection between miR-34a and aging signaling pathways, as shown in Fig. 4.

### miR Deregulates Proteostatis

**Figure 5.**
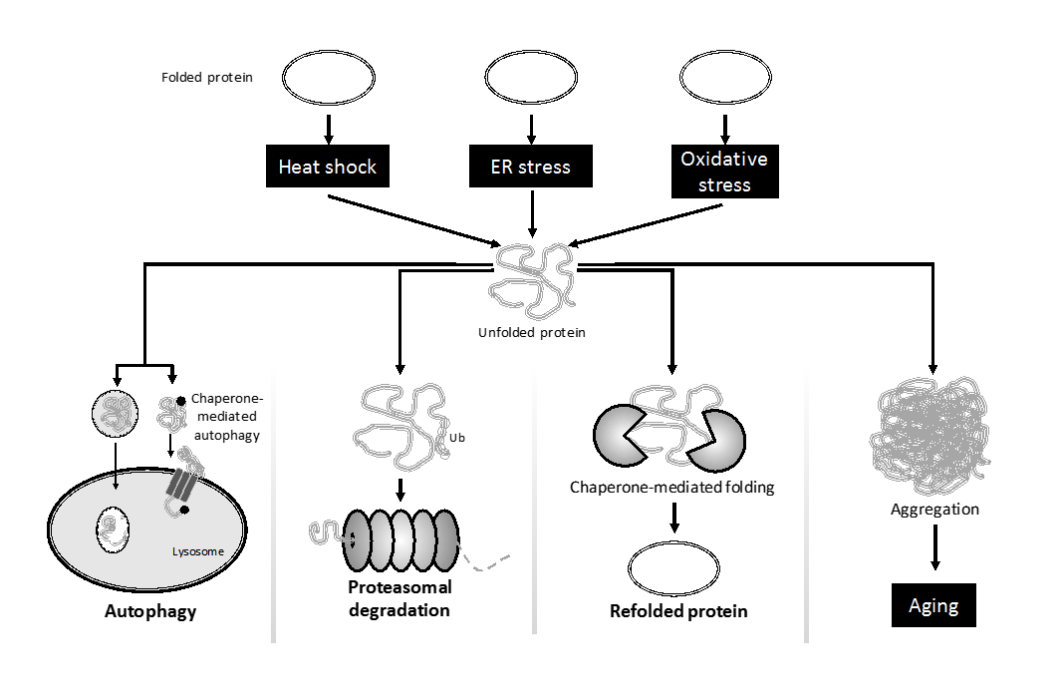
miRs specifically deregulate molecular chaperone specific pathway of proteostatis Oxidative and ER stress induced unfolded proteins either refolded back by HSP chaperone or degraded by HSC chaperone mediated ubiquitin-proteosome or lysomal pathways (Autophagy). Oxidative stress damages DNA and proteins leading to their reduced function. A miR-induced decrease in repair mechanisms would then encourage aging.

Proteostasis involves mechanisms for the stabilization of correctly folded proteins, as well as mechanisms for the degradation of misfolded proteins by proteasomes and lysosomes [33]. Impairment of proteostasis occurs in aging and in age-related diseases [34]. Chronic expression of unfolded, misfolded, or aggregated proteins is seen in age-related pathologies, including AD and Parkinson’s disease [35]. The genes, which are collectively called the molecular chaperones network, have a central role in proteostasis, as they are essential to preventing the accumulation of misfolded proteotoxic states, which commonly occurs in many neurodegenerative disorders [36]. Major players in the chaperone network include genes that encode heat shock proteins (HSP), HSP40, HSP20, HSP1OO, HSP110, HSP90, HSP72, and others [37]. As with many other cellular processes, miRs are also known to regulate the molecular chaperone network. The expression of miR-320 increases during cardiac injury, and has been shown to target HSP20 chaperone molecules, which is a cardioactive protein [38]. Increased expression of miR-106a, miR-26b, and miR-301b deregulates HSP70-mediated autophagy, and alpha-synuclein pathology in Parkinson’s disease [39]. In the aging heart [40] and in AD patients’ brain [41], increased expression of miR-34a and miR-146a has been reported. In silico analysis (Sarkar unpublished observation) identified HSP20, HSP40, HSP72 as targets of miR-34a and HSP90 as a target for miR-146a (Fig. 5).

### miRNAs and Autophagy

HSPs are not the only regulators of proteostasis; autophagy is another clearance system by which abnormal intracellular proteins are degraded. Recent studies have shown that miRNAs are involved in the induction of autophagy, as well as the formation of mature autophagosomes [42]. Autophagy consists of several sequential steps, and various miRs target many of the genes involved in these processes. First, autophagy induction is initiated by activation of the Unc-51 like autophagy activating kinase (ULK) complex, which is composed of ULK1/2, FIP200, ATG101 and ATG13 [43, 44]. It has been shown that miR-20a and miR-106b can directly target ULK1 and suppress its expression [45], while inhibition of miR-25 leads to autophagic cell death by directly increasing ULK1 expression [46]. Additionally, a recent study has reported that ULK2, an autophagy initiator, is a direct target of miR-885-3p [47].

miRNA-30a/b, miRNA-376b, miR-216a, and miR-17-5p inhibit Beclin-1 expression, which in turn suppresses initial stages of vesicle nucleation/autophagosome formation [48-51]. Beclin-1 can also be targeted by miR-519a, while miR-630 and miR-374a can inhibit UVRAG, which interacts with Beclin-1, leading to activation of autophagy [52]. Furthermore, it has been shown that miR-195 targets ATG14, a critical component of the class III PI3K/Beclin-1 complex for the nucleation of the autophagosomal membrane [53]. Vesicle nucleation/ autophagosome formation by RAB5A, a small GTPase, can induce autophagosome formation by its interaction with hVPS34 (a lipid kinase) and Beclin-1. miRNA-101 targets RAB5A to inhibit autophagy, implying that miR-101 modulates autophagy at the step of vesicle nucleation [54, 55]. Various miRNAs are also involved in the late stage process involving the retrieval and fusion by targeting the ATG2, ATG9, UVRAG and ATG18 genes [56-58].

### miRs Involved in Mitochondrial Dysfunction

Mitochondrial dysfunction is a feature of the aging brain and heart. Due to their high energy demand, these two organs are especially sensitive and vulnerable to any abnormality in mitochondrial function. Transcriptional profiling from individuals ranging from 26 to 106 years of age defines a set of genes important for mitochondrial function with severely reduced expression after age 40 [59]. These genes include ATP synthase, mitochondrial F1α, and nuclear encoded cytochrome c synthase, all of which are components of the electron transport chain. Dysfunction of the mitochondrial electron transport chain has also been associated with the pathophysiology of late-onset AD [60]. The most consistent defect in mitochondrial electron transport enzymes in AD is a deficiency in cytochrome c oxidase [61], which leads to an increase in ROS production, a reduction in energy stores, and a disturbance in energy metabolism [61]. Increased expression of various miRs in AD has previously been reported [62]. Two miRs, miR-34a and miR-146a, are of special interest because they target many of the electron transport chain genes. Severe reductions of electron transport chain proteins were observed in AD brains from human patients [41]. We also observed a severe reduction in mitochondrial oxidative phosphorylation and key electron transport proteins when miR-34a is over expressed in rat primary neurons [41].

### miRs and Stem Cells Exhaustion

Genes that regulate stem cell properties, so called “stemness genes”, control cell renewal, proliferation, and quiescence, and during aging, the function of these genes gradually declines. The high mobility group A2 (HMGA2) gene regulates stem cell pluripotency. Let-7 targets HMGA2 and increases p16 and p19 gene expression, thereby controlling stem cell aging [63]. Another miR, miR-145, can inhibit human embryonic stem cell differentiation by suppressing pluripotency genes, such as Oct4, Sox2, and KIf4 [64]. Many differentially expressed miRs, including let-7, miR-499, mir-371, and miR-369 in mesenchymal stem cells induce replicative senescence, which results in the loss of stemness [65]. miR-34a, b, and c suppress somatic cell reprogramming by repressing expression of the pluripotency genes Nanog, Sox2, and N-myc [66]. miR-720 also promotes differentiation by repressing the expression of the Nanog gene [67]. As such, loss of stemness during aging could arise due to the repression of pluripotency genes by miRs.

## 1. miRs Involved in Telomere Shortening, Cellular Senescence and Cardiac Aging

Aging in humans and mice is accompanied by progressive shortening of telomeres [68]. Telomere length is controlled in cells; shorter telomeres are associated with senescence, which can cause dysfunction in organs, while longer telomeres are associated with diseases such as cancer. Telomeres are maintained by the activity of a complex comprising of Telomerase Reverse Transcriptase (TERT), Telomerase RNA, Telomere Repeat binding Factor1&2 (TERF1,2), and Dyskerin Pseudouridine Synthase 1 [69]. Several miRs have been linked with changes in telomere length: miR-138 targets TERT transcript in a thyroid cancer cells [70]; miR-155 targets TERF1 [71]. It has also been shown that overexpression of miR-34a in gall bladder cancer tissues is associated with telomere shortening [72], and has been shown to be involved in apoptosis and senescence [73]. Recently, Boon et al., [40] demonstrated that miR-34a expression in human and mouse hearts increases in an age-dependent manner. Increased miR-34a expression in the aging mouse heart induces telomere attrition, causing cardiomyocyte cell death and inhibition of myocardial function. The authors identified a gene, PNUTS (synonym, protein phosphatase 1 regulatory subunit 10 (PPP1R10)), as a novel target of miR-34a, which is known to interact with TREF2 (Sarkar, unpublished data). Further, *in vivo* antagomiR-34a-mediated inhibition increases cardiac PNUTS levels, reduces cell death and fibrosis following acute myocardial infarction, and improves recovery of myocardial function [40]. In silico analysis (Sarkar, unpublished) revealed that miR-34a also targets TRET, and TREF2 genes. This downregulation may cause telomere shortening in the aging heart, which is currently associated with cellular senescence in cardiovascular cells, including cardiomyocytes, vascular endothelial cells, and vascular smooth muscle cells, resulting in cardiovascular diseases.

### miRs and Changes in Control Over Gene Expression

Molecular components that govern the structure and function of vital organs in the human body are encoded in the genome and regulated by the expression of various genes. As a consequence, changes in regulation of gene expression levels during aging might be directly related to the expression of the aging phenotype, and age-related pathology within a specific organ. GO and KREGG analyses of expression profiles of miRs in the aging cerebellum and cortex of chimpanzees and humans has shown that upregulated miR target genes are associated with cognitive decline and neurodegenerative disorders during aging [74]. There are various reasons for the changes in gene expression by miRs during aging, which includes alterations in the methylated states of regulatory DNA sequences, covalent modifications of histone proteins, changes in components of transcription machinery protein complex, and import/export defects in the nucleus.

### miRs and DNA methylation

Notably, miRs can cause aberrant DNA methylation. For example, the miR-29 gene family targets DNA methyl transferases, DNMT3A and DNMT3B, in cancer cells [75]. A conditional knockout of DNMT1 and DNMT3A in forebrain excitatory neurons of mice causes abnormal long-term synaptic plasticity in the hippocampal CA1 region, together with deficits in learning and memory [76]. In silico analysis revealed that miR-146a also targets DNMT3A (Sarkar, unpublished). miR-146a expression is increased in human late-onset AD brains compared to age-match controls [41].

### miRs and Histone Modification

Alterations in histone acetylation in animal models have been implicated in learning and memory, as well as synaptic plasticity. Studies based on pharmacological histone deacetylase (HDAC) inhibitors have primarily focused on class I HDAC family members (HDAC1, HDAC2, HDAC3, and HDAC8), with recent data suggesting that brain deletion of *Hdac2* facilitates memory formation and synaptic plasticity [77]. However, the role of class IIa HDACs (HDAC4, HDAC5, HDAC7, and HDAC9) [77], which act as transcriptional repressors and shuttle between the nucleus and cytoplasm in response to intracellular signaling, is not well known [78]. Recently it has been shown that selective loss of Hdac4 in the brain results in impairments in both hippocampal-dependent learning and memory, and long-term synaptic plasticity. In silico analysis indicates that miR-34a and miR-146a both target HDAC2 (Sarkar unpublished). As described earlier, expression of both miRs are increased in AD brain [41].

### miRs and Changes in Components of Transcription Machinery Protein Complex

One of the most crucial members of the transcription machinery protein complex is Topoisomerase II (TOP2). TOP2 removes torsional stress from chromosomal DNA and facilitates gene transcription by introducing transient DNA double-strand breaks (DSBs). Such DSBs are immediately rejoined by TDP2 gene encoding tyrosyl DNA phosphodiesterase-2, an enzyme that repairs TOP2-induced DSBs. It has been shown that neuronal activity triggers the formation of DNA double strand breaks in the promoters of many of the early response genes that are important for structural and functional synaptic plasticity and memory [79]. In silico analysis indicates that miR-34a directly targets both TOP2 and TDP2, as well as many immediate early genes, including the gene NPAS4 (Sarkar, unpublished). The NPAS4 gene is crucial for synaptic connections in excitatory and inhibitory neurons, neural circuit plasticity, and memory formation [80]. Besides immediate early genes, many other genes involved in synaptic plasticity and memory, whose expressions in human prefrontal cortex declines during aging, are also targets of miR-34a. Many synaptic plasticity genes show reduced protein expression associated with increased miR-34a expression [41]. In silico analysis also indicates that miR-34a targets the white matter plasticity gene, myelin regulatory factor (Myrf), which is a transcription factor, and the master regulator for myelination within the central nervous system (Sarkar, unpublished). Deletion of Myrf prevents mice from mastering complex motor learning tasks [81]. Thus, miR-34a targets many genes important for both grey and white matter plasticity and could therefore possibly impair cognitive function of the brain.

### miRs and Inflammation

Inflammation is an evolutionary adaptation designed to protect organisms from pathogenic infections. Resident tissue innate immune cells have pattern recognition receptors (PRRs) and are able to detect pathogen associated molecular patterns (PAMPs) expressed on microorganisms such as bacteria, and damage associated molecular patterns (DAMPs), which are altered or damaged endogenous ligands, usually released by necrotic, senescent, and/or damaged cells. Among PRRs, toll-like receptors (TLRs) play a central role, since their engagement activates a potent pro-inflammatory pathway, which is involved in signaling by NF-kB, mitogen activated protein kinases (MAPKs), and members of the interferon regulatory factors [82].

In addition to fighting off infections, inflammatory responses are generated to remove and restore damaged tissue after injury. For example, intrahippocampal injection of lipopolysaccharide (LPS) in amyloid precursor protein (APP) transgenic mice triggers an inflammatory response, resulting in transient clearance of diffuse amyloid-β (Aβ) deposits [83, 84]. Unfortunately, inflammation also has the capacity to damage healthy surrounding tissue as well [85]. Many believe that in AD, neuroinflammation is a driving force behind neurodegeneration [86, 87]. Cytokines expressed by activated glial cells or neurons may alter protein kinase pathways and tau phosphorylation patterns within the brain [88, 89]. The generation of reactive oxygen species (ROS) [90] and nitric oxide (NO) [91] may also lead to neuronal damage and subsequent death.

The body must be able to regulate and balance the immune response to allow for the clearance of pathogens and damaged tissue, while restricting the death of healthy cells. A mounting body of evidence documents a group of miRs that are involved in regulating inflammation. In normal physiological conditions, transcription of miR-165, miR-21 and miR-146a remains at low levels. However, initiation of TLR signaling results in strong co-induction and subsequent upregulation of these miRs, through pathways involving NF-κB signaling. It has been shown that depending upon both their target genes and affected tissue, miRs could play a dual function by inhibiting, as well as inducing, inflammation by TLR signals [92].

### The Blood-Brain Barrier Prevents Infiltration from the Periphery

The ways in which the CNS and the peripheral immune system influence one another has been heavily studied over the past few decades. As accumulating evidence supports intricate immune-nervous system interactions [93], we are beginning to understand how these two complex systems interact.

Unlike the vasculature in the periphery, the endothelial cells which comprise the BBB, form and maintain tight junctions in response to factors secreted by glial cells [94, 95]. In recent years, there has been a great deal of research focusing on the functional role of the BBB. Conclusive evidence shows that while tight junctions are selectively permeable and provide somewhat of a physical barrier, the BBB does not serve as an impenetrable blockade from all peripherally circulating factors.

Clinical and epidemiological evidence suggests that peripheral infection and systemic immune activation can lead to cognitive impairments in adults [96-98]. Work from animal models also suggests that systemic inflammation leads to increased hippocampal inflammation, behavioral deficits [99, 100], and AD-like pathology, such as tau hyperphosphorylation, increased Aβ42 accumulation and plaque formation, and learning and memory impairments [101-104].

During normal physiological conditions, tight junctions between endothelial cells prevent infiltration of unwanted cells, molecules, bacteria, and viruses into the CNS. It has been shown that both peripheral and neuroinflammation can weaken the BBB, allowing peripherally circulating factors, such as soluble, blood-derived plasma proteins [105], monocytes [106, 107], leukocytes [108-110], and pathogens to more readily enter the CNS [111] and thus, contribute to neuroinflammation. Peripherally circulating cytokines can induce upregulation of miR-155 in brain endothelial cells and lead to the disruption of tight junctions between cells [112]. Accumulating Aβ42 within the AD brain has been shown to reduce miR-107 in brain endothelial cells and lead to weakening of the BBB, the effects of which can be reversed upon overexpression of miR-107 [113]. Additionally, during periods of neuroinflammation, secretions from astrocytes can also weaken the BBB [114], as can miR-34a-induced mitochondrial dysfunction [115].

In order for immune cells to invade the CNS, they must first adhere to the brain endothelium; this is mediated by cellular adhesion molecules, namely ICAM-1 and VCAM-1 expressed on vascular endothelial cells (ECs). In response to TNF-α, IFN-γ, and IL-1, brain microvascular endothelial cells (ECs) upregulate ICAM-1 and VCAM-1 by 3- to 15-fold, thus enhancing immune cell invasion. These cytokines may play a large role in the infiltration of peripherally circulating leukocytes into the CNS [95], while also contributing to progression of the neuro-inflammatory response. Overexpressing miR-98 and let-7g* in brain microvascular endothelial cells reduces monocyte adhesion and decreases BBB permeability [116].

Aside from disease state, age can also heavily influence the permeability and functional capacity of the BBB [95, 111, 117]. Older adults and animals experience reduced transport of key metabolic molecules, such as glucose and choline, from the periphery into the CNS [118], which can lead to impaired neurological functioning. Just as in heightened inflammatory states, when the integrity of the BBB has been compromised with age, it becomes more permeable, or leaky, allowing peripheral substances entrance into the CNS [118].

Interestingly, there are areas of the CNS which lack a blood-brain barrier (BBB). Regions such as the circumventricular organs, ventricles, choroid plexus, and meninges lack tight junctions between the vascular endothelial cells; instead there are large fenestrations between cells which allow for direct communication between neurons and circulating blood [95, 119]. Not surprisingly, these areas elicit an immune response similar to what occurs in the periphery [93, 95].

### Contributions of Microglia in the Immune Response

When foreign, peripheral substances cross the BBB, microglia (which are commonly referred to as the resident macrophages of the CNS) become activated. Under normal physiological conditions, microglia were long believed to be resting, or quiescent; however evidence gathered over the past decade indicates that microglia are constantly surveying the microenvironment for disturbances, such as plaques, damaged neurons, alterations in extracellular potassium levels, or intruders like viruses or bacteria [120-124]. Like peripheral macrophages, microglia can engulf and eliminate cellular debris and present antigens to infiltrating T-cells [121].

Microglia play a pivotal role in the neuroinflammatory response [reviewed by 125]. When they become activated, they migrate towards the focal site of inflammation within minutes [126]. Although resting-state microglia constitutively express immune regulatory markers, their activation leads to a rapid upregulation of these cellular immune markers, such as complement receptors and major histocompatibility complex (MHC) II antigens [87]. Additionally, activated microglia have also been shown to secrete cytokines, chemokines, complement factors, and free radicals [127-129].

Microglia also help to regulate the neuroimmune response in chronic brain diseases, such as AD. For example, in the normal brain, APP is constantly being cleaved [87, 130], producing both soluble APPα (sAPPα) and sAPPβ [131]. Soluble APPα is non-pathogenic and does not accumulate, whereas sAPPβ can be processed to β-amyloid 1-42 (Aβ42), and aggregate into fibrils and plaques, characteristic of AD [131]. As extracellular Aβ42 accumulates and begins to form plaques, it attracts and activates microglia [132], which leads to the observation of a barrier-like ring of microglia around the plaque [133-135]. Aβ resides in the center of the plaque, and other molecules, such as complement factors, have also been observed within plaques [136]. Microglia express complement C3a receptors (C3aR) and can become activated upon receptor binding [137]. Complement-containing plaques are capable of inducing full blown complement cascades within the AD brain [136, 138, 139], contributing to chronic local inflammation.

Additionally, intracellular accumulations of Aβ42 have been observed within microglia [140], as Aβ42 molecules can be phagocytosed by microglia prior to aggregation in a dose- and time-dependent manner [141]. In healthy brains, this process occurs constantly [142], however in disease states, aggregated Aβ can be engulfed but not easily broken down, as it can remain un-degraded within the microglia for several days [143].

The neuroimmune response is a highly complicated and dynamic process. Aside from traditional immune interactions described above, miRs have also been recently identified to play a role in microglial activation and regulation in response to inflammation. Understanding how miRs play a substantial role in altering microglial activation, cytokine secretion, and overall activity is an important factor for understanding how neurodegenerative diseases progress. For a complete review on this subject, see [144].

Briefly, activation of microglia to either the “pro-inflammatory” M1 (classical activation) or “anti-inflammatory” M2 (alternative activation) phenotype appears to be dependent on a network of miRs. For example, LPS exposure shifts microglia towards the M1 phenotype, downregulates miR-Let-7a expression, and subsequently leads to cell death [145]. Overexpression of microglial miR-Let-7a altered the phenotypic activation to M2, reduced apoptosis and nitrite production, increased secretion of anti-inflammatory cytokines, and enhanced production of brain-derived neurotrophic factor (BNDF) [145]. Interestingly, miR-124 is highly expressed in resting-state microglia but becomes significantly downregulated when microglia take on the M2 phenotype [146]. Overexpressing miR-124 in microglia prevents microglial activation from even occurring [146].

Furthermore, miR-155 is classically regarded as a pro-inflammatory miR, and is upregulated in M1 activated microglia after exposure to cytokines like TNF-α and IFN-γ [147]. This has been shown to further enhance cytokine release from microglial cells; however, it has also been shown to induce IFN-β secretion, which appears to activate an anti-inflammatory pathway [148, 149]. These data suggest that miR-155 may influence early induction of the inflammatory response, while also acting to regulate it downstream.

As previously discussed, inflammaging is a concept in which the immune system shifts towards a pro-inflammatory state over the course of aging. Interestingly, yet not unsurprisingly, inflammaging seems to influence microglia and their capacity to react properly to brain insult. Microglia undergo a shift towards a pro-inflammatory state, and contribute to dysfunctional or exaggerated responses to inflammation, thus creating an unfavorable environment for neurons [as reviewed by 150, 151]. Studies investigating the protein and RNA profiles of microglia in mice at different ages indicate that age and sex do appear to play a role in expression patterns, and that polarization towards the M1 phenotype occurs at 12 months of age [152]. Furthermore, as reviewed by Spittau [153], several “hallmarks” of aging microglia have been described, including upregulation of TLRs, pro-inflammatory cytokines, and increased reactivity upon stimulation. However, it should be mentioned that M1 and M2 activation of microglia is not absolute; many studies have found microglial activation to be on more of a spectrum as opposed to two extreme activation states [154, 155], and has led to the generation of many opinion pieces voicing the same thoughts [156, 157].

By altering activation phenotype, alleviating cell death, or preventing microglial activation altogether, miRs have been implicated as potential therapeutic targets for inflammatory diseases of the central nervous system. Work in this area is ongoing, however many scientists appear to draw conclusions from studies of miR influences on macrophage activation and extrapolate these data to microglia as well [158-160]. Although both cell types are involved in the immune response, they are inherently different, and studies need to replicate findings from macrophages in microglia before any conclusive interpretations can be drawn.

### Aging and Inflammation

Human aging is characterized by chronic, low-grade systemic inflammation, which occurs during physiological aging in the absence of overt infection; this phenomenon has been termed as “inflammaging.” [161]. Inflammaging is a highly significant risk factor for the development and progression of age-related conditions, including cardiovascular disease, type 2 diabetes mellitus and neurodegenerative diseases [161]. The inflammatory response comprises complex biological reactions that require a fine-tuned integration between a range of immune system cells and an extensive network of biomolecules, which until recently had been thought to be largely cytokines. However, identification of a vast repertoire of miRs in the mammalian genome has completely revolutionized our understanding of most cellular processes, including inflammation.

Age-associated chronic inflammation has also been attributed to the accumulation of senescent cells with a pro-inflammatory secretory phenotype [162]. Induced expression of miR-146a in human umbilical and coronary endothelium during replicative senescence indicates that TLR/NF-κB activation and cell senescence can be modulated by miR-146a [163]. Arterial inflammaging highly contributes to cardiovascular morbidity and mortality. miR-34a has been implicated in tissue aging and endothelial progenitor cell senescence [164]. Furthermore, miR34a induces vascular smooth muscle cellular senescence in aortas of old mice by inducing the transcriptional expression of a subset of pro-inflammatory factors [32].

### miRs and Neurons

Neuronal growth and differentiation relies heavily on proper miR signaling pathways. For example, in order for newly born neurons to mature and differentiate into granule cells within the hippocampus, miR-132 is required for proper dendritic formation [165]. Further discussion of miR roles in neuronal development and maturation is beyond the scope of this review, but these topics have been previously reviewed elsewhere [166, 167].

miRs have been implemented in the formation of memories, which relies heavily on hippocampal synaptic plasticity. Cognitive impairments such as diminished memory formation and recollection are among the first symptoms observed in many neurodegenerative diseases such as mild cognitive impairment (MCI) and AD, and can be implemented in other diseases like Parkinson’s disease (PD) and multiple sclerosis (MS) at a later stage of disease progression.

In order for long-term memories to be created, alterations in the synapse must occur. These changes require the presence of readily available proteins, or protein synthesis at or near the synapse; this requires the presence of mRNA. The presence of mRNAs encoding for local protein synthesis (as opposed to somatic protein synthesis, followed by transport to the synapse) is now widely accepted [extensively reviewed in 168].

Within the last decade, evidence has surfaced that miRs targeting mRNA of important synaptic forming proteins are present in dendritic spines. As such, if protein translation is being repressed by miRs within a post-synaptic density, then proper synaptic change will not occur, leading to impaired memory formation [169]. Limk1 is a protein kinase present in dendritic spines that controls their development [170]. miR-134 has been shown to negatively regulate *Limk1* mRNA translation at the synapse; until the appropriate external stimulus is received by the neuron, Limk1 protein synthesis is repressed [170].

### miRs and Brain Diseases/Injury

Many diseases that plague our society stem from yet unknown etiologies. Recent developments in human genomics better allow us to understand disease-related alterations in DNA, RNA, and proteins at a molecular level. The newly emerging field of bioinformatics sheds a great deal of light on the regulation of miRs and the proteins they effect. Understanding how levels of specific miRs, or clusters of miRs, change in response to various stressors, inflammatory markers, and disease states could provide promising potential therapeutic targets for future medical implementation. A large portion of work regarding miRs and disease focuses on characterizing miR profiles; for example, identifying and understanding miR panels prior to the onset and during the progression of AD. Identifying a panel of miRs and their gene targets could illuminate specific pathways that are implicated in disease development and progression, allowing us to better understand specific diseases and create novel therapeutics for them.

### miRs and Alzheimer’s Disease

AD is a neurodegenerative disease characterized by memory impairment, confusion, anxiety, and depression, and is evidenced by brain atrophy, ventricle enlargement, Aβ42 plaques, and hyperphosphorylated tau. Presently, it is the 6^th^ leading cause of death in the United States and there is no cure or effective treatment for the disease [https://www.alz.org/facts/; 171]. The cause of this disease and the driving force behind its progression appears to be very complex and may rely heavily on genetic risk factors and regulatory mechanisms, which may easily implicate miRs in disease onset and progression.

AD is thought to be preceded by MCI. It has been widely shown that adults diagnosed with MCI experience chronic brain vasculature hypoperfusion, which can lead to increases in Aβ deposits and death receptor-6 (DR6) protein, activation of caspases-3 and -6, and dendrite degeneration [172]. The presence of DR6 has been implicated with neuronal loss and dendritic degeneration. miR-195 targets DR6 mRNA; upregulation of this miR has been demonstrated to reduce translation of DR6 protein, and subsequently diminish the loss of both dendrites and neurons [172]. AD-linked neuronal loss and impaired neurite differentiation has also been linked to upregulation of miR-211-5p; and these effects were found to be exacerbated in the presence of extracellular Aβ [173].

miRs have been shown to influence the expression and aggregation of the brain-specific microtubule-associated protein, tau. Both clinical evidence and transgenic animal models indicate that anxiety is a common psychiatric symptom experienced in AD [174, 175], and it is likely due to tau dysregulation [176]. Anxiety may be the result of upregulated miR-92a in AD brains; one target of this miR is vGAT, which is responsible for loading GABA, the main inhibitory neurotransmitter, into secretory vesicles [177]. Dysregulation of GABA signaling has been thought to contribute to anxiety, as neurons are left in an unregulated, overexcited state. Both the overexpression of vGAT and the administration of a miR-92a antagomir reduced anxiety-related behaviors in a mouse model of overexpressed human tau [177], suggesting a crucial role for miR-92a in the GABA secretory pathway, with subsequent effects on anxiety behavior.

Our lab has recently demonstrated that miR-34a is significantly upregulated within the temporal cortex of post-mortem AD brains, as well as the temporal cortices and hippocampi of a triple transgenic mouse model of AD (3xTgAD) [41]. Additionally, overexpression of miR-34a in primary neurons leads to subsequent mitochondrial dysfunction, as indicated by reduced mitochondrial respiration and ATP production [41]. A key observation in AD patients is a reduction in glucose metabolism; abhorrent upregulation of miR-34a may be a main contributor to this observed phenomenon within the hippocampus.

miRs such as miR-132 and miR-212 have been implicated in the regulatory capacity of neuronal nitric oxide synthase (NOS1) in humans; these miRs are downregulated in AD and other tauopathies [178]. Due to the downregulation of miR-132 and miR-212, NOS1 is increased in these diseases and activates a deleterious cascade, which results in tau hyperphosphoyrlation and neuronal cell death [178]. Secretion of miR-132 in neuron-derived exosomes to endothelial cells regulates vascular endothelial cadherin (VE-cadherin/Cdh5) protein expression by targeting *eukaryotic elongation factor 2 kinase* (*eef2k* ) [179]. In circumstances where levels of miR-132 are decreased, Cdh5 protein levels decrease, and BBB integrity becomes compromised [179].

In search of a potential biomarker for AD, many groups have profiled miR expression from patients’ blood and blood components (plasma and serum) [reviewed by 180] and post-mortem brains [reviewed by 181]. Across multiple investigators and sample types, the most consistently identified miRs seem to be miR-9, miR-125b, miR-39, miR-155, and miR-146a. Identifying biomarkers of AD has great potential for disease diagnostics, however it becomes very complicated when levels of miRs change so drastically across sample types, detection methods, and labs. For example, while one group observed a downregulation of miR-9 in AD serum when compared to controls, using qRT-PCR [182], another group observed miR-9 upregulation in AD serum compared to controls, also using qRT-PCR [183]. Differential patterns in miR expression convolute the ability to identify one or several definite miR biomarkers for non-invasively detecting AD. However, it is clear that miR expression is altered in AD when compared to age-matched controls, indicating that miRs must be influencing disease progression in some manner.

### miRs and Parkinson’s Disease

Parkinson’s disease (PD) is a progressive neurological disorder that affects movement in its early stages, and cognitive function in later stages [184]. The etiology of the disease is not well understood, however like most neurodegenerative diseases it is thought that the interplay between genetics and environmental factors leads to disease development. In PD, neurons within the substantia nigra die, and physical symptoms such as tremors or rigidity occur. As the disease progresses, patients often experience depression and anxiety, sleep disturbances, and dementia.

The family of miR-29s (miR-29a, b, and c) was found to be significantly downregulated in PD patients when compared to controls; these miRs have been implicated in neuronal proliferation, differentiation, plasticity, and survival, and their downregulated expression may lead to subsequent loss of neuronal viability in PD patients [185].

In addition to miR-29, miR-1, miR-22, miR-133b, and miR-433 have also been found to be significantly downregulated in PD patients when compared to controls [186-188]. Tubulin polymerization-promoting protein (TPPP/p25) is enriched in neurological brain lesions and its accumulation may contribute to alpha-synuclein aggregation in PD brains; miR-1 targets TPPP/p25, therefore reductions of miR-1 can lead to pathological overexpression of TPPP/p25 and subsequent alpha-synuclein aggregates [186]. In an *in vitro* cell culture model of PD, miR-22 was shown to be downregulated in PD-induced cells, and was associated with cell death and stunted cell proliferation; overexpression of miR-22 attenuated these effects and enhanced cellular survival and proliferation [189].

Ceruloplasmin is a key enzyme in regulating iron metabolism. In PD, it has been shown to be significantly reduced in serum and CSF compared to healthy controls; its reduced presence may enhance the deposition of iron in the substantia nigra of PD patients [190]. Recent work suggests that miR-133b downregulation modulates *ATP7B* gene expression, and indirectly alters ceruloplasmin levels leading to subsequent nigral iron deposition [190]. Work from other labs however does not reveal differential expression of miR-133b in substantia nigra dopamine neurons when compared to age-matched controls [191], or in dopaminergic neuron development [192], so further work is required to parse out the potential influences of miR-133b in PD. Furthering this point, another lab studied miR-433 variations in PD patients and found no differences from control patients [193].

In PD patients, compared to controls, has-miR-4639-5p was found to be significantly upregulated, leading to downregulation of DJ-1 protein which has been shown to cause oxidative stress and cell death [194]. The commonly used model of induced PD is animals exposed to 1-methyl-4-phenyl-1,2,3,6-tetrahydropyridine (MPTP). *In vivo* experiments have shown that after PD induction, miR-124 levels are significantly reduced, and these results were also observed in an *in vitro* MPTP-induced PD induced model [195]. Subsequent analysis indicated that miR-124 may play a protective role as its knockdown leads to increased ROS and H_2_O_2_ production and enhances expression of the calpain 1/p25/cdk5 protein pathway, leading to neuronal cell death [195]. Additionally, miR-181c has been identified as significantly downregulated in blood and brain samples of PD patients. In an *in vitro* model of PD, this dysregulation was also observed and was associated with neuronal apoptosis, ROS, and caspcase-3 activation [196].

### miRs and Stroke

Strokes occur when a cerebral artery is blocked (ischemic stroke; 87% of cases) or ruptures (intracerebral or subarachnoid hemorrhage; 10 and 3% of cases, respectively), resulting in a loss of blood flow to the brain area supplied by that artery [197]. Stroke is the 5^th^ leading cause of death, and a leading cause of long-term disability, with limited treatment options. Although proven effective if administered to an eligible patient population, the therapeutic window of recombinant tissue plasminogen activator (tPA), the most widely adopted thrombolytic agent, is exceptionally short (3-4.5 hours from symptom onset), due to the increased risk of cerebral hemorrhage when given after that time point. There is a critical need to develop rapid and accurate diagnostic tools, as well as novel therapeutic interventions for the treatment of ischemia; miRs hold great promise for both.

During stroke, as cells deprived of oxygen and glucose begin to die, an inflammatory response is initiated within the brain. In response, activated microglia begin secreting many factors, some of which prompt further neuronal cell death, such as TNF-α and IL-1β [198]. As TNF-α upregulation occurs, there is a pronounced downregulation of miR-181c in activated rat microglial cells; this relationship can be reversed by transfecting microglia with miR-181c, thus decreasing levels of TNF-α, reducing inflammation, and subsequently reducing neuronal cell death [198]. Interestingly, another group found increases in levels of all miRs of the 181 family (a-d) post-stroke within the ischemic core, but decreases in these same miRs in the penumbra region [199]. By knocking down levels of miR-181a, cell death in response to oxygen/glucose deprivation was reduced [199]. Similarly, in male rats subjected to bilateral carotid artery occlusion, miR-181a levels were increased relative to sham animals; treatment of stroke animals with a miR-181a antagomir increased Bcl-2 levels and decreased neuronal loss [200]. Microglial activation, and subsequent cytokine release, has been shown to be inhibited by knockdown of miR-377 [201]. Targets of this miR include EGR2 and VEGF, which influence inflammation and angiogenesis, respectively. In a rat MCAO stroke model, knocking down miR-377 reduced ischemic infarct size by reducing inflammation mediated by microglial activation, and promoting angiogenesis, thus leading to better stroke outcomes [201].

Additionally, favorable stroke outcomes rely partially on the integrity of the BBB post-insult. Recent data implicate significant post-stroke decreases of miR-122 in whole blood of both stroke patients and animal models, and the possibility that this miR plays a role in mediating stroke outcomes [202]. Targets of miR-122 include proteins that affect cell adhesion and leukocyte extravasation. When miR-122 was administered into the blood, lower infarct volume and better neurological outcomes were observed as compared to when it was directly injected into brain ventricles, indicating that this miR is likely affecting CNS leukocyte invasion via peripheral mechanisms rather than directly influencing CNS tissue [202]. In the stroke-prone spontaneously hypertensive rat model, it was shown that prior to onset of stroke, levels of miR-122 within the brain are decreased substantially. Additionally, vascular endothelial cells showed heightened levels of inflammation and death, leading to degradation of the BBB, however these responses were attenuated after exposure to miR-122 [203]. Together, these data suggest that miR-122 acts through the blood to improve the integrity of endothelial cells comprising the BBB.

In the same vein, miR-126 and miR-126* also appear to regulate leukocyte adhesion and infiltration across the BBB [204]. These miRs are downregulated in response to pro-inflammatory cytokines, such as TNF-α, which tend to be upregulated in disease states. When they are downregulated, VCAM1 and E-selectin levels are increased, respectively, and leukocyte adhesion significantly increases [204], which can lead to enhanced leukocyte extravasation into the brain, thus worsening patient outcomes. Additionally, miR-126 has been shown to facilitate vascular remodeling after cardiovascular insult, such as stroke. In a mouse line with conditional knock-out of miR-126 in endothelial cells, knockout mice fared worse than controls post-stroke, exhibiting higher levels of inflammation and cardiac hypertrophy [205]. Stroke-induced miR-126 downregulation appears to play a large role in outcomes related to inflammation and overall cardiovascular health after insult.

Other examples of miRs thought to have important roles in mediating ischemic stroke outcomes include miR-146a/miR-146b [206], miR-9 [207], and miR-130b [208]. Upregulating the expression levels of miR-146a and miR-146b was shown to promote proliferation and angiogenesis of endothelial progenitor cells after acute ischemic stroke in mice [206]. While not yet directly linked to stroke, miR-9 has been shown to enhance neural stem cell proliferation, as well as angiogenesis, which could potentially work to enhance post-stroke CNS repair [207]. Interestingly, miR-130b exerts its protective effects by regulating aquaporin 4 (AQP4) in astrocytes [208]. Levels of AQP4 have been shown to be significantly upregulated in astrocytes post-stroke and are associated with enhanced cerebral edema [209]. Post stroke levels of miR-130b are significantly reduced, and continue to decrease over 24-48 hours, allowing for increased translation of AQP4 [208]. By transfecting primary astrocytes with miR-130b mimics, the deleterious effects of oxygen/glucose deprivation (OGD) were attenuated and expression of AQP4 was inhibited [208]. Additionally, miR-29 appears to be associated with cell survival/death pathways. miR-29b levels are increased at 24 hours following 90 min tMCAO in rat brains [210] and found to promote cell death. In rats, miR-29c overexpression reduced anti-apoptotic proteins, increased infarct, and negatively impacted neurological outcomes [211]. Interestingly, in mice, miR-29b [212] and miR-29c [213] levels are reduced in brain and overexpression reduces lesion volume, edema, blood brain barrier disruption, and cell death.

The severity of stroke outcomes also relies on the extent of injury caused by cerebral reperfusion after blood flow has been restored. miR-323 has been shown to be upregulated in response to OGD in a cell model of ischemic stroke and is associated with apoptosis by reducing levels of Brain protein I 3 (BRI3) [214]. In response to stroke, miR-30a has been shown to be upregulated within the infarct core, but downregulated in the peri-infarct area after reperfusion, which appears to be neuroprotective [215]. miR-30a targets the mRNA of Beclin-1, which is a protein that mediates neuronal autophagy; when levels of Beclin-1 are decreased, neuronal death is enhanced as the cells are unable to adequately degrade damaged internal molecules created in response to OGD [215].

After cerebral ischemia, expression of miR-124 is significantly downregulated in the brain [216]. With constant intracerebroventricular infusion of a miR-124-antagomir prior to MCAO, animals had better neurological outcomes post-stroke, and smaller infarct areas [216]. These data indicate that miR-124 may be an important player in reducing negative stroke outcomes in both mice [146, 217, 218] and rats [216]. However, other reports indicate that in mice experiencing tMCAO, brain miR-124 levels increased following stroke and experimentally-induced overexpression was associated with neuroprotective effects and beneficial outcomes [219, 220]. These conflicting reports exemplify the complexity of miR-mRNA interactions in response to ischemic stroke.

With regard to sex, it is as yet unclear what role it plays in miR changes in brain trauma. Work done by the Sohrabji and McCullough groups has noted a key role of sex and sex hormones in the context of experimental stroke. For example, sex differences have been noted for post-stroke brain levels of miR-23a, a target of which is X-linked inhibitor of apoptosis (XIAP), such that miR-23 was decreased in males but increased in females [221]. Interestingly, inhibition of XIAP significantly increased infarct volume in females but not males, providing evidence for sex-specific effects of stroke on miRs. As well, antagomir treatment of miR-1 and -Let7f, miRs that target the IGF-1 pathway, was beneficial among adult female, gonadally intact rats but not males or ovariectomized females [222]. It should be noted that the majority of studies assessing miR level changes in stroke are conducted in male rodents and in many reports, there was no documentation of, nor control for, endogenous reproductive hormone levels, a potential critical biological factor given the known neuroprotective actions of estrogen and progesterone in the context of stroke and other CNS pathologies. Future investigations specifically considering sex as a biological variable in the miR-associated response in stroke are warranted.

Despite this, miRs have great potential to serve as easily accessible peripheral biomarkers for stroke [223, 224]. In preclinical studies where miRs were evaluated in both brain and peripheral tissues following stroke, while tissue specific changes in some miRs were noted, others were similarly dysregulated in both brain and blood [225, 226]. The ability to differentiate brain injury type and stage, especially when the etiology of injury or stage of progression is unknown, is of important clinical relevance to the treatment of stroke given the limited time window with which tPA can be administered following an ischemic event, and given that it is contraindicated for hemorrhagic stroke [227]. This could potentially allow for rapid, accurate diagnostics when stroke patients enter the emergency department as opposed to requiring lengthy, expensive imaging techniques to confirm stroke occurrence. Unfortunately, as is the case in AD, different labs identify varying miR profiles. These differences could be due to a number of factors which could influence relative amounts of miRs detected in patient samples, including the location of the stroke, the length of time that passes between symptom onset and sample collection, treatment interventions, and the health status/history of the patient.

### miR Disease Therapeutics

As alluded to in each of the preceding sections discussing miRs and specific diseases, scientists and clinicians are heavily invested in the promise of using miRs as potential biomarkers for particular diseases. Initially, miRs seem like promising candidates for disease identification because of their relative stability in the blood [228], as bound by lipoproteins and ribonucleoprotein complexes, or encapsulated within microvesicles and exosomes [229, 230], preventing their degradation by RNases. However, the issue of utilizing this small RNA species as a biomarker from disease arises in the heterogeneity within an individual disease, and across hospitals and laboratories. These confounding findings could be due to a number of variables such as timing of sample collection in regards to disease progression and therapeutic interventions, RNA isolation techniques, and quantification methods. More stringent guidelines are required from the scientific community before differential findings can be viewed as solely disease-relevant variations.

Aside from biomarkers, the use of miRs has been viewed as potential therapeutic strategies. Personalized medicine is seeing more and more discussion within the medical field, and identifying genetic variations caused by diseases could allow for direct genetic targeting in medicine. For example, a patient with AD, found to overexpress a specific miR, could be administered an antagonist for the miR in question. Conversely, if a miR was observed in significantly decreased levels, the patient could be supplemented with synthetic oligonucleotide strands to mimic the miR.

An alternative approach for keeping overexpressed miRs in check would be to focus on miR recognition elements (MREs) on target mRNA, which can sponge miRs, therefore preventing their interaction with functional mRNA molecules [231, 232]. For example, ribonuclease P RNA Component H1 (*Rpph1* ) competes with *CDC42* (a Rho GTPase which enhances dendritic spine formation) for miR-326-3p/miR-330-5p. Overexpression of *Rpph1* results in increased dendritic spine formation, as more CDC42 is able to be translated [232]. Theoretically, this intervention strategy could be applied in diseases characterized by reductions in dendritic spines, thus enhancing synaptic plasticity.

These types of disease interventions are currently in the early stages of exploration [233], however it is a very complex therapeutic strategy and will most likely take years to develop efficient and safe treatment methods. A main issue facing the field is that many of the predicted targets of altered miRs are currently unknown, and it is important to remember that a single miR can target many different mRNAs, and a single mRNA can be targeted by many different miRs. Preventing the aberrant function of a specific miR implicated in a disease-related pathway may indeed alleviate the issue associated with the targeted cascade, however, because of their complexity, other miR-mRNA interactions may be subsequently altered and have deleterious off-target effects.

Lastly, evaluating miR changes in age-related neurological conditions should be assessed in the context of the treatment regimens typically employed and known to induce neurobeneficial effects (i.e. tPA and stroke), as these considerations will be critical to understanding the cellular mechanisms of actions of current therapeutic treatments, and developing more specific interventions. Despite these remaining challenges, as we have discussed above within several neurological disease conditions, several patterns in alterations of miRs have emerged that may represent viable candidates for use as biomarkers for injury severity and/or disease progression, and possibly serve as novel therapeutic targets towards which neurorestorative treatments can be directed. Future research will continue to clarify these remaining questions.

### Conclusion and Future Perspectives

Extensive research to identify functional roles of miR target genes has provided insights into the molecular mechanisms by which aging processes are regulated at the cell, tissue, organ, and organism level. Further, determination of aging-related miRs have yielded a better understanding of the connection between the processes of aging and the onset of aging-related diseases. Aging phenotypes are known to be interconnected; for example, telomere attrition is a characteristic of cellular senescence, which is connected to activation of the DNA damage response and acquired senescence associated secretory phenotype (SASP). Likewise, a number of miRs are regulators of overlapping hallmarks of aging, shown in Figure 1. Recent studies have also indicated that a single miR, miR-34a, correlates with aging of both mice and humans; affording the consideration of miR-34a to be a biomarker for the aging brain and heart.

In the aging heart, miR-34a induces telomere attrition, DNA damage responses, cardiomyocyte apoptosis, and deteriorates recovery after acute myocardial infarction. In aging brains and in the brains of patients with AD, miR-34a targets genes involved in neural activity-dependent grey and white matter plasticity, activity-dependent angiogenesis, neurodegeneration, and memory impairment. Aging-induced heart and brain specific miRs, like miR-34a, may be involved in the pathogenesis of cardiovascular diseases and cognitive dysfunction. Although we still do not fully understand the molecular mechanisms by which aging induces miR expression in a specific tissue, it has been reported that human miR-34a promoter contains the p53, NF-kB, and STAT3 transcription factor binding sites [41], which can be activated in aging tissues due to increased ROS and inflammaging. Thus, molecules that alter the function or abundance of specific miRs e.g., miR-34a, represent a new target strategy for treating human diseases.

Recent progress in the development of effective strategies to block miRs suggest that anti-miR drugs may soon be used in the clinic. The major strategy for inhibiting miR function is the use of antisense oligonucleotides of mature miRs. Antisense oligonucleotides used in many preclinical stages are of three types: i), 2’-O-methyl (2’-Ome), ii) 2- methoxy methyl (2’MOE), iii) 2, 4-methyl locked nucleic acid (2-4-LNA). Currently, the development status of most miRNAs is at the preclinical and target peripheral diseases. miR-122 is being applied for Hepatitis C Virus (HCV), miR-21 for renal fibrosis, miR-33 for atherosclerosis, miR-92 for peripheral artery disease, and miR-15 for myocardial infarction, using antisense oligonucleotides for inhibition of respective miRs function. In initial clinical assessment of miR therapeutics, a miR-34a mimic is in a phase I trial for the treatment of primary liver cancer. Also, Santaris Pharma is studying miR-122 antisense oligo in a phase Ia clinical trial for the treatment of hepatitis C. A new strategy is miR sponge nanoparticles. The miR sponge nanoparticle downregulates the targeted miR and possesses multiple complementary sites to the targeted miRs [234]. Targeted delivery of anti-miR oligonucleotides remains difficult for many organs, including the brain. Thus, for effective and targeted delivery of anti-miR oligonucleotides to a specific tissue, new technologies are required.
